# Work stress and depressive symptoms in older employees: impact of national labour and social policies

**DOI:** 10.1186/1471-2458-13-1086

**Published:** 2013-11-21

**Authors:** Thorsten Lunau, Morten Wahrendorf, Nico Dragano, Johannes Siegrist

**Affiliations:** 1 Institute for Medical Sociology, Medical Faculty, Heinrich-Heine-University Düsseldorf, P.B.101007, 40001 Duesseldorf, Germany; 2 Senior Professorship on Work Stress Research, University of Duesseldorf, 40225 Duesseldorf, Germany; 3 International Centre for Life Course Studies in Society and Health (ICLS), Research Department of Epidemiology and Public Health, University College, London, UK

**Keywords:** Depressive symptoms, Labour and social policies, Work stress, Demand-control, Effort-reward imbalance, Cross-national study

## Abstract

**Background:**

Maintaining health and work ability among older employees is a primary target of national labour and social policies (NLSP) in Europe. Depression makes a significant contribution to early retirement, and chronic work-related stress is associated with elevated risks of depression. We test this latter association among older employees and explore to what extent indicators of distinct NLSP modify the association between work stress and depressive symptoms. We choose six indicators, classified in three categories: (1) investment in active labour market policies, (2) employment protection, (3) level of distributive justice.

**Methods:**

We use data from three longitudinal ageing studies (SHARE, HRS, ELSA) including 5650 men and women in 13 countries. Information on work stress (effort-reward imbalance, low work control) and depressive symptoms (CES-D, EURO-D) was obtained. Six NLSP indicators were selected from OECD databases. Associations of work stress (2004) with depressive symptoms (2006) and their modification by policy indicators were analysed using logistic multilevel models.

**Results:**

Risk of depressive symptoms at follow-up is higher among those experiencing effort-reward imbalance (OR: 1.55 95% CI 1.27-1.89) and low control (OR: 1.46 95% CI 1.19-1.79) at work. Interaction terms indicate a modifying effect of a majority of protective NLSP indicators on the strength of associations of effort - reward imbalance with depressive symptoms.

**Conclusions:**

Work stress is associated with elevated risk of prospective depressive symptoms among older employees from 13 European countries. Protective labour and social policies modify the strength of these associations. If further supported findings may have important policy implications.

## Background

Maintaining work ability among older employees is an important goal of national policies in rapidly ageing societies in Europe and beyond. Physical and mental health are key determinants of work ability [1,2]. In recent years, a growing impact of mental disorders, particularly depression, on work ability in terms of sick leave and disability was documented in several countries [3,4]. In addition to established risk factors of depression [5], exposure to a chronically stressful work environment increases the probability of developing depressive disorders, especially so if stressful work is measured by the demand-control or the effort-reward imbalance model [6,7]. The former concept posits that jobs defined by high demands in combination with low decision latitude or task control are stressful [8], whereas the latter model is based on the notion of failed contractual reciprocity between efforts spent and rewards received at work, where rewards include money, promotion prospects, job security, and esteem [9]. Taken together, both work stress models cover different, but equally relevant aspects of the workplace, where lack of control and reward frustration matter most. It is of theoretical and practical interest to know whether distinct national labour and social policies have an influence on work related health problems. Conceptually, these policies may be of importance in at least two ways: First, they may exert an influence on the prevalence of a stressful work environment. Second, they can modify the effect of stressful work on health and well-being [10]. The first assumption has been supported previously and existing evidence (based on comparative European data) indicates that national active labour market policies (ALMP) are related to better working conditions, in particular those policies that promote further education and workplace training among older people [11,12]. However, the evidence for the second assumption is still limited [13-15]. This limitation is partly due to a lack of cross-national studies, but also to the problem of how to define and measure relevant aspects of labour and social policies. While ALMP may promote psychosocial working conditions in general, one may assume that their impact on the health-adverse consequences may be different. For instance, aspects of employment protection may be more important in this case.

In this contribution we set out to overcome these limitations by studying the following research questions: (1) Do we observe significant associations of indicators of a stressful work environment with depressive symptoms across a variety of countries (12 European countries and the United States)? (2) Can we observe protective effects of distinct favourable national labour and social policies on the strength of this association (modification of effect of stressful work on depressive symptoms)?

With the first research question we address the paucity of available cross-national studies, in particular longitudinal investigations as the one reported here. With the second research question we propose to focus on core aspects of national labour and social policies which may represent protective resources in our context (see below). Rather than relying on established typologies of national welfare regimes [16,17] we maintain that the following more specific policy measures are better suited to reflect protective policy effects: (1) the amount of the state’s investments in active labour market policies (ALMP), (2) the degree of employment protection provided by the state, and (3) the degree of distributive justice as reflected, e.g. in the amount of income inequality.

The first measure may protect workers against the threat of being excluded from a core social role in adult life [18], whereas the second measure protects those who are at risk of being excluded due to job loss [19]. With the third measure a relevant collective sense of fairness is identified which may mitigate stressful experience of inequity at work [20]. All three aspects of national labour and social policies exert their effect on wellbeing of employees by reducing the amount of threat experienced in case of job instability, forced retirement, major income shocks, degradation, or loss of job autonomy. In terms of theories of stressful experience, these threats to occupational status affect workers mental and physical health as they undermine essential feelings of continued control and reward at work [21]. As the notions of control and reward are embedded in the two work stress models mentioned, our conceptual approach enables us to link distinct macro-structural contexts with individual-level experience of work and health (for measurement see Methods).

Impact of stressful work on depressive symptoms as well as potential protective resources provided by national labour and social policies are of particular relevance in view of an ageing workforce, as mentioned above. Therefore in this contribution we analyse our two research questions by referring to three longitudinal surveys of older employees (50 to 64 years) with similar study design and well comparable measurements of core variables.

## Methods

### Data

Data were obtained from three longitudinal ageing studies, ‘the Survey of Health, Ageing and Retirement in Europe’ with information on 11 European countries (SHARE, Release 2.3.0), the English Longitudinal Study of Ageing (ELSA, Release 2) and the US Health and Retirement Study (HRS, 2004 Final V1.0, HRS 2006 Final V2.0, RAND Version J). Details on each survey are provided elsewhere [22-25]. To allow cross-national comparisons all studies were developed in close coordination. Countries range from Scandinavia (Denmark and Sweden), England, Central Europe (Austria, France, Germany, Switzerland, Belgium, and the Netherlands), Southern Europe (Spain, Italy and Greece) to the United States. The studies are based on representative samples of individuals aged 50 and older with ongoing waves of data collection in two-year intervals covering a variety of sociological, economic and health-related topics. Participants were interviewed using Computer Assisted Personal Interviews (CAPI) and self-completion questionnaires. We used data from two waves, collected in 2004 and 2006. In the SHARE study the average household response rate in 2004 was 61.6%, ranging from 39% (Switzerland) to 81% (France). The individual response rate range from 74% in Spain to 93% in France. In ELSA and HRS the response rates in 2004 are 82% and 87.8%. The sample is restricted to men and women aged 50–64 years reporting to do any paid work in 2004. Moreover, to study new incidences of clinically relevant depressive symptoms between both waves, all participants with increased depressive symptoms in 2004 were excluded. This restriction results in a total sample of 5650 participants with full available data.

### Measurement

Work stress: In all three studies work stress was assessed by short versions of validated scales. Given the constraints of a multi-disciplinary approach in the three studies the inclusion of full original questionnaires was not possible. Thus, items measuring the theoretical core dimension of the two work stress models were selected on the basis of factor loadings on respective original scales. With regard to the demand-control model, the measurement was restricted to the control dimension as core dimension of the demand-control model. Low control at work was measured by the sum score of two Likert-scale items, with higher scores indicating lower control at work [8]. For each country, participants scoring in the upper tertile of the score were considered to experience stressful work in terms of low control [26]. To measure effort-reward imbalance, 2 items measuring ‘effort’, and 5 items assessing ‘reward’ at work were included. This selection was based either on the original questionnaire [9] or on its abbreviated, psychometrically validated version [27]. For the selected items, all item-total correlations were far beyond the established threshold of 0.30 [28] ranging from 0.93 to 0.81 (uncorrected) and from 0.67 to 0.42 (corrected). ‘Effort-reward imbalance’ was defined by a ratio of the sum score of the two scales, adjusted for unequal number of items, where country specific tertiles were calculated [9]. Values in the upper tertile were defined as exposure to psychosocial stress at work in terms of this latter model [26]. A summary of all items is presented in an additional table (Additional file 1).

Depressive symptoms: To measure depressive symptoms, binary indicators of a clinically relevant mental state were defined on the basis of two internationally established instruments, a short form of the Centre for Epidemiologic Studies Depression (CES-D) scale (8 items) [29], and the EURO-D depression scale [30] (12 items). The former scale was applied in the ELSA and HRS studies, and the latter scale in the SHARE study. A high degree of comparability of results of the two scales was demonstrated [30,31]. Cut points (≥ 4) of the scales indicating clinically relevant depressive symptoms were validated by clinical interviews [32,33].

Additional measures: In addition to sex and age (3 categories), education, income, employment status, work time, functional limitation (at least one limitation in activities of daily living) and several self-reported chronic conditions (stroke, high blood pressure, diabetes, heart disease) were considered as confounders. Annual household income was categorized into country-specific tertiles. For SHARE and ELSA education was measured according to ISCED-97. In the HRS study corresponding levels were obtained based on years of education due to the fact that ISCED-97 is not available in the HRS study [34]. Finally, type of employment (self-employed vs employed) and work time (full-time (<35 hours per week) versus part-time) were measured.

Macro-structural indicators: Six macro indicators were selected from OECD online databases [35,36]. The first three indicators represent the dimension of the state’s active labour market policy, indicators four and five capture the degree of employment protection by the state, and indicator six measures the level of distributive justice in terms of income distribution. The six indicators are as follows: (1) the overall level of ALMP expenditures (percentage of gross domestic product (GDP)); (2) the amount of investments in rehabilitation services (percentage of GDP); (3) the extent to which older working people (55+) participate in continued learning (participation rate); (4) the extent of income maintenance and support for unemployed persons (percentage of GDP); (5) the degree of union density (percentage of workers belonging to any trade union); (6) the degree of income inequality measured by the Gini coefficient.

### Statistical analyses

After a basic sample description and bivariate analyses, logistic regression models are calculated to estimate odds ratios of developing depressive symptoms between 2004 and 2006. Given the multilevel structure of the data, we apply multilevel methods with individuals (level 1) nested within countries (level 2) [37]. In doing so the dependence of residuals within a country is considered since the constant is allowed to vary across countries. The constant consists of a fixed part and a random error term for each single country. As a consequence, the standard deviation of this error term (sigma u in Tables) informs about between-country variations of the constant. Thus, its proportion of the total variance can be estimated (rho). Following our first research question, we first estimate odds ratios of depressive symptoms for each of the two work stress models separately. In doing so, the distinct effect for each measure can be estimated and compared [38] (though correlation between the two measures was rather low (Phi = 0.24)). Odds ratios are adjusted for all covariates mentioned above. In the results, we present estimated odds ratios and confidence intervals. In addition, variability parameters between countries are shown for the random component (sigma u, rho), and the log likelihood, the AIC (Akaike Information Criterion), and the BIC (Bayesian Information Criterion) statistics are indicated.

**Figure 1:**
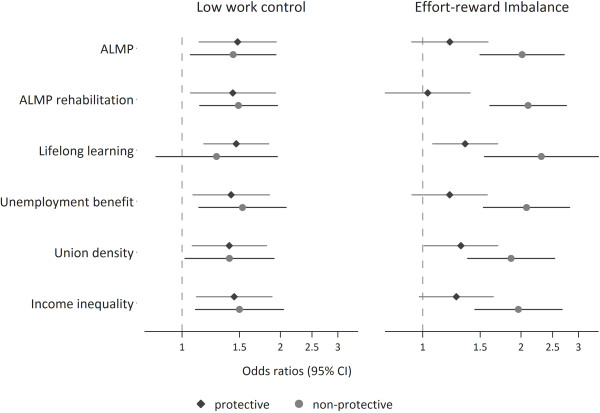
**Associations of work stress and elevated depressive symptoms at follow up stratified by policy context (protective vs. non-protective).** Results of multilevel models (odds ratios and 95% confidence intervals). Test of modification of the effect of work stress on depressive symptoms by six labour/social policy indicators is presented in Table 4.

To test the second hypothesis, modification of the effect of work stress on depressive symptoms by the macro indicators, we first explore associations between work stress and depressive symptoms according to the macro indicators within stratified analyses. More specifically, each macro indicator was dichotomized, based on the respective country rank order, and was labelled ‘protective’ or ‘non-protective’ accordingly (e.g. protective ALMP versus non-protective ALMP). On this basis, Figure 1 presents odds ratios estimated for both macro-groups separately, and thus, allows to compare visually the strengths of associations between protective and non-protective policies (for each of the six macro indicators and for both work stress models). To allow for precise comparisons of odds ratios between protective and non-protective groups, we retain the symmetry of odd ratios and present them on a logarithmic scale [39]. Finally, as a formal test of effect-modification, we test the significance of interactions between work stress and the dichotomised macro indicators (e.g. effort-reward imbalance * non-protective ALMP) in non-stratified analyses. In the results, we present main effects of work stress and the interactions as odds ratios with confidence levels (95%). In doing so, the interactions indicate whether (and to what extent) effect sizes differ between the two contexts [40]. All calculations were done using STATA 11.

HRS was approved by the institutional review board from the University of Michigan Health Services. SHARE was approved by the institutional review board at University of Mannheim, Germany. Ethical approval for ELSA was obtained from the Multi-Centre Research Ethics Committees in the United Kingdom.

## Results

An overview of the longitudinal sample is presented in Table 1 together with the percentage of people developing increased depressive symptoms between 2004 and 2006. The total sample consist of slightly more men (3176) compared to women (2474) and the large majority (81%) was younger than 60 at baseline assessment. Variations of depressive symptoms are found for sex (higher among women), age (higher among younger people), socioeconomic position (higher among those in lower positions), work time (higher among those working part time), self-reported chronic conditions (higher among persons with stroke and diabetes) and functional limitations (higher among persons with at least one limitation in activities of daily living). Moreover, we observe a higher percentage of depressive symptoms among employees experiencing work-related stress compared to the remaining group.

**Table 1:** Description of measures and sample (N = 5650)

**Variable**	**Categories**	**(%)**	**N**	**% of depressive symptoms**	**P-value**
Sex	Male	56.2	3176	6.2	
	Female	43.8	2474	11.4	.000
Age group (2004)	50-54 years	40.1	2263	9.6	
	55-59 years	40.8	2304	8.4	
	60-64 years	19.2	1083	6.2	.004
Effort-reward Imbalance	Yes	30.0	1696	11.1	
	No	70.0	3954	7.3	.000
Low work control	Yes	29.8	1683	11.0	
	No	70.2	3967	7.4	.000
Income	Low	31.0	1744	11.0	
	Medium	34.1	1926	9.9	
	High	35.0	1980	7.1	.000
Education	Low	26.8	1512	9.5	
	Medium	38.0	2144	8.9	
	High	35.3	1994	7.1	.023
Employment status	Self-employed	17.2	970	7.2	
	Employed	82.8	4680	8.7	.126
Work time	Part-time	26.9	1517	9.6	
	Full-time	73.2	4113	8.1	.072
Heart disease	Yes	6.5	367	9.3	
	No	93.5	5283	8.4	.567
High blood pressure	Yes	25.0	1414	8.8	
	No	75.0	4236	8.4	.629
Stroke	Yes	0.9	51	19.6	
	No	99.1	5599	8.4	.004
Diabetes	Yes	5.0	281	11.0	
	No	95.0	5369	8.3	.112
≥1 Limitation in activities of daily living	Yes	3.0	170	14.7	
	No	67.0	5480	8.3	.003
Country	Sweden	11.7	660	9.6	
	Denmark	6.4	360	9.7	
	Germany	6.2	349	11.8	
	Netherlands	7.2	407	6.4	
	Belgium	8.3	468	11.3	
	France	5.7	321	13.7	
	Switzerland	3.5	195	7.2	
	Austria	3.0	168	8.3	
	Italy	3.6	204	15.7	
	Spain	3.1	174	13.2	
	Greece	7.0	397	1.8	
	England	24.1	1360	5.8	
	USA	10.4	587	8.0	.000
Total			5650	8.5	

Note. P-values are based on chi-square tests.

These findings were confirmed in multivariate analyses, as presented in Table 2. The risk of experiencing newly manifested depressive symptoms at follow-up is significantly higher among women, in the younger age group, among persons with limitations of daily living, among persons who had a stroke and in the low income group. Importantly, in case of both models of work stress we see strong associations between work stress and incident depressive symptoms (Hypotheses 1).

**Table 2:** Associations of work stress with risk of elevated depressive symptoms at follow up: results of multilevel estimates (odds ratios and 95% confidence intervals)

	**Incident depressive symptoms (assessed in 2006) (N = 5650)**		
		**Effort-reward imbalance**	**Low work control**
Fixed parameters			
Effort-reward Imbalance	Yes	1.55 (1.27-1.89)	
	No(Ref.)		
Low work control	Yes		1.46 (1.19-1.79)
	No(Ref.)		
Sex	Female	2.01 (1.63-2.48)	2.00 (1.63-2.47)
	Male (Ref.)		
Age group (2004)	50-54 years	1.49 (1.11-2.00)	1.50 (1.11-2.01)
	55-59 years	1.30 (0.96-1.75)	1.31 (0.97-1.76)
	60-64 years (Ref.)		
Income	Low	1.50 (1.15-1.95)	1.48 (1.14-1.93)
	Medium	1.35 (1.08-1.69)	1.35 (1.08-1.69)
	High (Ref.)		
Education	Low	1.13 (0.87-1.47)	1.09 (0.84-1.43)
	Medium	1.11 (0.87-1.40)	1.09 (0.86-1.38)
	High (Ref.)		
Employment status	Employed	1.04 (0.79-1.37)	1.00 (0.76-1.31)
	Self-employed (Ref.)		
Work time	Full-time	0.99 (0.79-1.25)	1.05 (0.84-1.32)
	Part-time (Ref.)		
Heart disease	Yes	1.29 (0.88-1.90)	1.31 (0.89-1.92)
	No (Ref.)		
High blood pressure	Yes	1.05 (0.84-1.32)	1.04 (0.83-1.30)
	No (Ref.)		
Stroke	Yes	2.73 (1.32-5.67)	2.76 (1.33-5.72)
	No (Ref.)		
Diabetes	Yes	1.41 (0.94-2.11)	1.41 (0.94-2.11)
	No (Ref.)		
≥1 Limitation in activities of daily living	Yes	1.88 (1.19-2.96)	1.94 (1.23-3.05)
	No (Ref.)		
Random parameters			
Sigma u		0.46 (0.28-0.76)	0.46 (0.28-0.76)
Rho		0.06	0.06
Log likelihood		−1558.22	−1560.86
BIC		3263.31	3268.58
AIC		3150.44	3155.71

In addition, Table 2 displays significant, but small between-country variations of depressive symptoms, with an intra-class correlation (‘rho’) of 0.06. This indicated that only 6% of the total variations in depressive symptoms are related to differences between countries, and that – vice versa - most of the variations are related to differences between individuals.

To answer our second research question (effect-modification) Table 3 first displays the distribution of the macro indicators under study. Concerning investments into ALMP, rehabilitative services and income maintenance, we observe some convergence between the three indicators within single countries or among groups of countries with similar social and labour policies (e.g. high ALMP expenditures in Scandinavian countries, low expenditures in England and the USA). A similar pattern is observed with regard to union density, with the exception of England. While these findings partly correspond to existing welfare state regimes (e.g. ‘social-democratic’ versus ‘liberal’), [16] less consistent associations are observed regarding ‘lifelong learning’ and ‘income inequality’. (e. g. relatively high income inequality in Germany or France, i.e. in countries not traditionally considered ‘liberal’ welfare states).

**Table 3:** Labour/Social policy indicators by country (rank order)

**Country**	**ALMP**^**a**^	**Rehabilitative services**^**b**^	**Lifelong learning**^**c**^	**Unemployment benefit**^**d**^	**Union density**^**e**^	**GINI**^**f**^
Sweden	0.98 (2)	0.22 (4)	61 (1)	1.29 (7)	78.1 (1)	0.24 (2)
Denmark	1.37 (1)	0.30 (2)	29 (5)	1.94 (3)	71.7 (2)	0.23 (1)
Germany	0.84 (5)	0.15 (5)	28 (6)	2.27 (1)	22.2 (8)	0.30 (5)
Netherlands	0.89 (3)	0.56 (1)	29 (5)	2.09 (2)	21.3 (9)	0.27 (3)
Belgium	0.87 (4)	0.12 (6)	29 (5)	1.56 (5)	53.1 (3)	0.27 (3)
France	0.72 (6)	0.06 (8)	16 (9)	1.63 (4)	7.8 (13)	0.28 (4)
Switzerland	0.64 (7)	0.25 (3)	45 (2)	1.03 (9)	19.6 (10)	0.27 (3)
Austria	0.44 (10)	0.04 (9)	25 (7)	1.12 (8)	34.4 (4)	0.27 (3)
Italy	0.54 (9)	0.00 (12)	12 (10)	0.64 (10)	34.1 (5)	0.35 (8)
Spain	0.63 (8)	0.07 (7)	17 (8)	1.46 (6)	15.5 (11)	0.31 (6)
Greece	0.14 (11)	0.00 (12)	5 (11)	0.40 (11)	24.5 (7)	0.31 (6)
England	0.06 (13)	0.01 (11)	37 (4)	0.19 (13)	29.4 (6)	0.34 (7)
USA	0.11 (12)	0.03 (10)	40 (3)	0.27 (12)	12.0 (12)	0.37 (9)

^a^ALMP: expenditure in % of GDP invested into active labour market programmes in 2004.

^b^Rehabilitative services: expenditure in % of GDP invested into rehabilitative services in 2004.

^c^Lifelong Learning: participation rates in workplace training or education for persons aged 55 to 64 in 2007.

^d^Unemployment benefit: expenditure in % of GDP invested into income maintenance and support in 2004.

^e^Union density: percentage of salary earners that are trade union members in 2004.

^f^GINI: measure of inequality of income (mid 2000′s).

Source: OECD [35,36].

In Figure 1 we can explore visually the different effect sizes of work stress on depressive symptoms by the six macro indicators – each dichotomized and labelled ‘protective’ or ‘non-protective’ as described in the method section. In case of low work control odds ratios are generally similar in the two groups. In contrast, we see that the effect sizes between effort-reward imbalance and depressive symptoms are generally stronger in a ‘non-protective’ policy context. Finally, to formally test whether effect size are significantly different between protective and non-protective contexts, we additionally calculated main effects of work stress on depressive symptoms together with interaction terms. Results are given in Table 4 showing estimated odds ratios for the main effects of work stress and interactions. The interaction term indicates whether (and to what extent) the association is significantly higher in the group of participants working in a ‘non-protective’ policy context. For instance, in case of effort-reward imbalance the interaction ‘Poor working conditions * Low ALMP’ means that the odds ratio in the ‘non-protective’ policy context (OR 2.13) is 1.77 higher compared to the ‘protective’ policy context (OR 1.20) [40]. In case of the effort-reward imbalance model four out of six interaction terms are statistically significant and the main effects are non-significant in these latter cases. This indicates that health-adverse effects of work stress are restricted to ‘non-protective’ contexts and supports the notion of a modifying effect of distinct ‘protective’ policies on the strength of associations of work stress with depressive symptoms. The hypotheses were not supported for ‘union density’ and ‘lifelong learning’. In case of low control at work no significant interactions are observed, though, main effects are significant in five of six cases. In other words, effects of low control on depressive symptoms are observed in all cases, with no differences between protective and non-protective contexts.

**Table 4:** Modification of the effect of work stress on depressive symptoms by six labour/Social policy indicators

	**Effort-reward imbalance**	**Low work control**
Poor working conditions (main effect)	1.20 (0.92-1.57)	1.37 (1.05-1.81)
Poor working conditions * Low ALMP	1.77 (1.19-2.64)	1.14 (0.76-1.71)
Poor working conditions (main effect)	1.03 (0.77-1.39)	1.32 (0.97-1.77)
Poor working conditions * Low ALMP rehabilitation	2.13 (1.43-3.18)	1.22 (0.81-1.82)
Poor working conditions (main effect)	1.39 (1.11-1.75)	1.50 (1.19-1.89)
Poor working conditions * Low Lifelong learning	1.55 (0.98-2.45)	0.89 (0.56-1.43)
Poor working conditions (main effect)	1.21 (0.93-1.57)	1.36 (1.03-1.78)
Poor working conditions * Low unemployment benefit	1.80 (1.20-2.68)	1.18 (0.79-1.78)
Poor working conditions (main effect)	1.34 (1.03-1.74)	1.48 (1.13-1.93)
Poor working conditions * Low union density	1.40 (0.94-2.09)	0.97 (0.64-1.45)
Poor working conditions (main effect)	1.26 (0.97-1.63)	1.35 (1.03-1.76)
Poor working conditions * High income inequality	1.67 (1.12-2.49)	1.21 (0.81-1.82)

Note. Multilevel logistic regression analysis with interaction terms (Poor working conditions * labour/social policy indicators) and main effects (odds ratios and 95% confidence intervals N = 5650).

Odds ratios are adjusted for age, sex, education, income, employment status, work time, heart disease, diabetes, stroke, blood pressure and activities of daily living.

## Discussion

This study provides new evidence on two research questions. First, in a cross-national study including 5650 working men and women aged 50 to 64 years from 13 countries, we find significantly increased odds ratios of depressive symptoms at two-year follow-up among participants experiencing work-related stress in terms of effort-reward imbalance and low job control. Effects based on multilevel analysis are adjusted for relevant confounders.

Second, in case of the effort-reward imbalance model, four out of six indicators of a ‘non-protective’ labour or social policy at national level modify the effect of work stress on depressive symptoms, with significantly stronger odds ratios compared to those observed in ‘protective’ policy contexts. Importantly, to our knowledge, this is the first study demonstrating that distinct labour and social policies can buffer the effect of work stress on depressive symptoms. The health threatening stress response of effort-reward imbalance might be less pronounced if effort-reward imbalance is experienced in a ‘protective’ policy context. The reward dimension of the effort-reward imbalance concept consist of the subdimensions ‘esteem’, ‘salary’, ‘job promotion and job security’. One may assume for example that the threatening effect of job insecurity is less severe if protective policies (e.g. unemployment benefit) exist. In case of low control main effects on depressive symptoms were significant, with no differences by contexts (no significant interactions). One interpretation is that the effect of low control is rather independent of the contexts, and that – while important for the general level of low control (e.g. [11,12]) – national policies matter less once a person is exposed to adverse working conditions.

It may be premature to interpret these findings in the frame of a protective role of distinct welfare state policies on the working populations health and well being. Yet, it is important to note that the effort-reward imbalance model puts its focus on threats to the work role in terms of low wage or salary, low esteem or appreciation, poor promotion prospects and low job security in response to high efforts spent at work. These threats are particularly harmful if experienced under challenging macro-economic conditions of elevated levels of unemployment, forced job mobility and wage cuts [41,42].

This study has several limitations. First, in an effort to compare data obtained from 3 surveys of older employees covering 13 countries, the available measures of our core variables represent short versions of original scales and, in case of depressive symptoms, are restricted to standardized self-assessed questionnaires. Despite satisfying psychometric properties of these scales, improved measurements [3] are recommended for future studies. For example the studies used in our analyses only include the control scale of the demand control model. Second, the availability of macro-structural labour and social policy indicators at national level was restricted to easily accessible online data bases provided by OECD. These indicators are still relatively crude, and quality of data may vary to some extent across countries. In addition, the number of countries included in this study is still relatively small although they represent a fair spectrum of economically advanced nations. Third, we lost a fraction of our sample due to non-response and missing data (effort-reward imbalance 8.0%; low control 4.8%; depressive symptoms 1.8%). However, additional analyses revealed only minor evidence of systematic bias. It should also be mentioned that the sample size in some countries was rather small, thus limiting the robustness of some analyses.

One general problem of longitudinal surveys is sample attrition. In the SHARE study the attrition rate between wave 1 and wave 2 is 27.9% [43]. ELSA and HRS have lower attrition rates. This could have affected our results. However, the attrition rate was only slightly higher for employees with low control and there was no higher attrition rate for people with effort-reward imbalance. These limitations are balanced by several strengths. First, we were able to use comparable standardized measures of main variables of interest taken from three pioneering epidemiological studies on economic, social and health-related characteristics of ageing populations in Northern, Western and Southern Europe and the United States of America, SHARE, ELSA, and HRS. The respective sample was large enough to conduct multivariate statistical analyses with appropriate confounder control. Second, consistent associations of two theoretically grounded measures of work-related psychosocial stress with newly occurring depressive symptoms were observed. Third, we applied an innovative approach towards estimating a modifying role of national welfare policies by selecting three types of macro-structural indicators reflecting (1) active labour market policies, (2) employment protection by the state, and (3) distributive justice in terms of income inequality. These indicators are thought to represent protective resources against the threats of psychosocial stress at work, thus mitigating adverse effects on workers’ mental health [11].

In view of the challenging occupational public health problem of depression in rapidly ageing societies the findings of this study, if supported by further evidence, may have important policy implications. Improved efforts of national labour and social policies to provide protective resources against the threats of stressful work to their workforce can contribute to a reduction of harmful effects on their mental health, especially so in times of unrestrained neoliberal policies and related financial crises.

## Conclusion

In conclusion, this study demonstrates that stressful work is associated with an elevated risk of depressive symptoms in a large sample of older employees in 13 economically advanced countries. Moreover, indicators of national labour and social policies were shown to modify the effects of work stress in terms of effort-reward imbalance on depressive symptoms. Findings lend support to policy efforts towards increasing investments into good quality of work and employment.

## Competing interests

The authors declare that they have no competing interests.

## Authors’ contributions

All authors read and approved the final manuscript. TL, MW and JS drafted the manuscript. TL and MW conducted the empirical data analysis. ND contributed to the study design, revised the manuscript and contributed to its final version.

## Pre-publication history

The pre-publication history for this paper can be accessed here:

http://www.biomedcentral.com/1471-2458/13/1086/prepub

## Supplementary Material

Additional file 1 Effort-reward imbalance and low control items. Click here for file
