# Supplementary material for: Work stress and depressive symptoms in older employees: impact of national labour and social policies
Source: BMC Public Health. 2013 Nov 21;13:1086. doi: 10.1186/1471-2458-13-1086 (PMC4222833; doi:10.1186/1471-2458-13-1086)
Supplement: Additional file 1 — Effort-reward imbalance and low control items. [file 1471-2458-13-1086-S1.pdf]

Additional file 1: Effort-reward imbalance and low control items

I am now going to read some statements people might use to describe their work. We would like to know if you feel like this about your present job. Thinking about your present job please tell me whether you strongly agree, agree, disagree or strongly disagree with each statement.

Effort-reward imbalance		Low control
**Effort**	• My job is physically demanding<br>• I am under constant time pressure due to a heavy workload	• I have very little freedom to decide how I do my work<br>• I have an opportunity to develop new skills
**Reward**	• I receive the recognition I deserve for my work<br>• Considering all my efforts and achievements, my salary/earnings is/are adequate<br>• My job promotion prospects/prospects for job advancement are poor<br>• My job security is poor<br>• I receive adequate support in difficult situations.	
